# The Impact of Depression on the Functional Outcome of the Elderly Stroke Victim from a Gender Perspective: A Systematic Review

**DOI:** 10.3390/healthcare10102110

**Published:** 2022-10-21

**Authors:** María Salud Franco-Urbano, María del Carmen Rodríguez-Martínez, Patricia García-Pérez

**Affiliations:** 1 Occupational Therapy. Centro de Día CITEA. C/Virgen de Montserrat, 10, 41011 Sevilla, Spain; 2 Department of Physiotherapy, Faculty of Health Sciences, University of Málaga, C/Arquitecto Francisco Peñalosa, 3, 29071 Málaga, Spain; 3 Occupational Therapy Department, Hospital Universitario Virgen de la Victoria, Servicio Andaluz de Salud (SAS), 29010 Málaga, Spain; 4 Faculty of Medicine, University of Málaga, 29010 Málaga, Spain

**Keywords:** stroke, depression, treatment outcome, quality of life

## Abstract

(1) Background: The aim of this systematic review focused on analyzing the impact of depression on the functional outcome of the elderly stroke victim and how this disorder affects both the female and the male population. (2) Methods: We conducted a systematic review using the Preferred Reporting Items for Systematic Reviews and Meta-Analyses (PRISMA) guidelines. The review was registered in PROSPERO (ID 346284). The systematic search for clinical trials was performed in the databases Pubmed, Otseeker, Scopus, Web of Science, Psycinfo, Medline Complete, ScienceDirect, SciELO, and Dialnet. Articles were selected according to the inclusion and exclusion criteria, including those dealing with post-stroke depression in adults whose psychological status had changed. Studies that only assessed the psychological state of caregivers were excluded. (3) Results: In total, 609 articles were identified, of which 11 randomized controlled trials were finally included in the review. The results indicate that post-stroke depression influences the recovery of functionality and quality of life. In addition, the need to detect the mood of the adult population after the stroke and to provide individualized treatment according to the characteristics of the person is highlighted. (4) Conclusions: This systematic review shows how early detection of post-stroke depressive symptoms can improve the degree of disability and quality of life of the person, especially in women.

## 1. Introduction

Cerebrovascular Accident (CVA), also known as stroke, occurs when the blood vessels leading to the brain are affected. The World Health Organization (WHO) considers this pathology to be the second leading cause of death in the world and the third leading cause of physical disability in adults [1].

The World Stroke Organization (WSO) declares that 15 million people suffer a stroke each year and six million of whom do not survive the episode [2]. In Spain, the prevalence of people who have suffered a stroke is estimated to be approximately 7% of the population over 65 years of age. This figure is expected to increase by 35% by 2035 [3].

According to the “IBERICTUS” study by Díaz-Guzmán et al. [4] carried out in Spain in 2006, 10% of strokes occur in young people and 54% in women. It is established as the first cause of death in women and the third in men, with greater severity, mortality, and worse evolution in women.

Two out of every three people who survive a stroke have some type of sequelae, which can even be disabling [5]. This event is frequently associated with depression [6]. It was found that 59% of patients who have had a stroke have problems performing ADLs, 62% have mobility problems, 64% suffer pain and discomfort and 36% perceive their state of health as poor. These percentages have a significant impact on the family and community. Stroke represents a heavy economic burden (an estimated 6 billion euros per year, 5% of Spanish public health expenditure) [7].

The impact of stroke will depend on some factors such as social or injury-related factors, altering occupations, and life satisfaction. Risk factors are classified as modifiable (e.g., lifestyle) and non-modifiable (e.g., biological conditions) [8].

The decrease in a person’s functional capacity does not only occur at the time of the accident, but the person also faces various physical and psychosocial challenges after discharge from the hospital. This whole process of change leads to an increased risk of chronic disability along with depression and social isolation [9]. Depression is present in 40% of people one year after a stroke, which can affect their quality of life and the recovery process. The social impact and the probability of suffering depression are greater in women due to the fact that they are older at the time of the stroke and more likely to live alone or isolated [10].

Depression is a common disorder affecting 3.8% of the population, 5.7% of whom are adults over 60 years old. It is the result of the interaction between social, psychological, and biological aspects. Those who experience adverse circumstances are more likely to suffer from depression. This disorder can cause increased stress and it becomes a serious health problem when it is recurrent and of moderate-severe intensity [11].

Beck (1964) considers depression as a negative interpretation of oneself and one’s experiences. Cognitive theory defines human depression as a sense of personal failure to control life events leading to a state of frustration [12].

Depression has a negative impact on the quality of life of the person suffering from it, experiencing a loss of interest in occupations. There is a close relationship between depression and physical health. The performance decreases with depression and the psychomotor and sensory-perceptual areas are affected, resulting in possible language disorders and reducing executive functions such as memory [13].

Differences between men and women in relation to stroke are increasingly recognized, not only in terms of risk but also in terms of the influence of stroke etiology, symptoms, and outcomes [14].

Between the ages of 45–54 years, the incidence in women increases as menopause begins and their hormone levels decrease. From the age of 55 years, the incidence of stroke is comparable between women and men until the age of 85 years or older, at which age the higher risk of stroke (especially ischemic) in women is confirmed [15]. Women have worse functionality before stroke, multimorbidity, and lower social support. This contributes to gender and age differences [16].

The differences between women and men are attributed to the longer life expectancy of the female sex and age, which is a strong risk factor and, in turn, a prognostic factor for negative clinical outcomes [17].

Women have a higher lifetime risk of stroke due to higher mortality, disability, depression, and dementia after a first stroke [18]. A prospective study on gender and stress in predicting depressive symptoms after stroke by Mazure et al. [19] found that the severity of depressive symptoms was higher in women than in men and they also increased in women due to stress.

Finally, one study about the impact of gender on stroke pathology and treatment by C. Gibson and Attwood [15] confirms that the incidence of thrombotic stroke is more frequent in men, while embolic strokes are more frequent in women. This last part is evidenced by a study of how some factors affect post-stroke depression López-Espuela et al. [20] which relates mood to the type of stroke. Moreover, it states with certainty that the most depressed are those who suffer a total infarction of the anterior circulation producing dysphasia, which is related to stroke in women, as an artery to the brain is blocked and produces the same oral affectation [20].

### 1.1. Problem Statement

Post-stroke depression is the most frequent disorder and the main factor limiting patients’ recovery and rehabilitation [7,8,9,10,11,12]. It occurs in one out of three stroke patients and more than half go undiagnosed and consequently untreated [13,14]. The study “Secondary Prevention of Small Subcortical Strokes” examined the prevalence of depression and it was associated with lower occurrence in older males without cognitive impairment [21,22].

It is difficult to distinguish those signs and symptoms that belong to stroke or depression [23]. Some symptoms are common or not recognized as consequent to this mental disorder and the diagnosis is underestimated [24]. In addition, questions remain as to what the best time is to initiate treatment and whether the only use of antidepressants has a direct effect on cognition and motor function [7,17]. These two areas, together with effectivity, play an important role in the impact of cerebrovascular disease.

Beyond pharmacological treatment, a rehabilitation program is necessary to recover functional capacity, independence, and reintegration of the individual into his or her family, social and work environment. Rehabilitation is adjusted to the level of disability, sequelae, and severity of the individual. If it starts in the first weeks, it leads to better long-term results [8,23,24].

### 1.2. Importance of Occupational Therapy

Stroke can be addressed by the clinical practice of Occupational Therapy at both physical and psychological levels to achieve the promotion of people’s health, prevention of permanent disability status, and participation in the environment towards engagement in occupation [25,26,27,28].

The framework for Occupational Therapy practice defines the characteristics that make up the client as a dynamic interaction between psychological aspects, bodily functions, and the surrounding environment. The balance in these three aspects determines the commitment toward optimal occupational performance [29].

The illness process and the adaptation to a new situation can lead to a deficit in performance skills, loss of old habits, routines or roles, and alteration of the contexts and environments in which the individual used to relate [29].

Occupational therapy theory and practice can create a person-centered clinical rationale to support the recovery process. It provides the opportunity to develop a plan according to the client’s needs, to adapt the rehabilitation process, and to offer counseling guidelines, both in the hospital setting and in the personal environment [29].

The lack of an Occupational Therapy service in the hospital context leads to late detection of functionality and psychological aspects such as depression suffered in the disease process [30].

This service can provide an opportunity for a type of intervention such as a counseling process to work with the clients to identify their problems, consider possible solutions, and modify the new situation they are facing [31].

Therefore, Occupational Therapists can assist other professionals in the early detection of post-stroke depression and they can offer different intervention approaches to prevent the disability and the reduced functionality that is perceived in people who suffer a stroke causing them long-term problems and high healthcare costs [24,25] So, the main objective of this systematic review is to analyze the effect of depression on the functional outcome and long-term recovery of patients who have suffered a stroke.

In turn, two secondary objectives are set: to analyze the impact of this mental disorder in the female versus male elderly population; to highlight the need for occupational therapy to address both the physical and psychological rehabilitation of this pathology during the period of admission and discharge.

## 2. Materials and Methods

The development of this systematic review was guided by the “PRISMA 2020” statement and its registration information is available [32].

### 2.1. Information Sources

This systematic review was conducted through an advanced search between February and April 2022 in the following databases: ‘Pubmed’, ‘Scopus’, ‘Web of Science’, ‘Psycinfo’, ‘Medline Complete’, ‘Science Direct’, ‘OTseeker’, ‘SciELO’ and ‘Dialnet’ (see Table 1). At this stage, it was checked that no other review had been carried out on the same topic. Then, the registration was completed in PROSPERO (ID 346284).

### 2.2. Search Strategy

**Figure 1:**
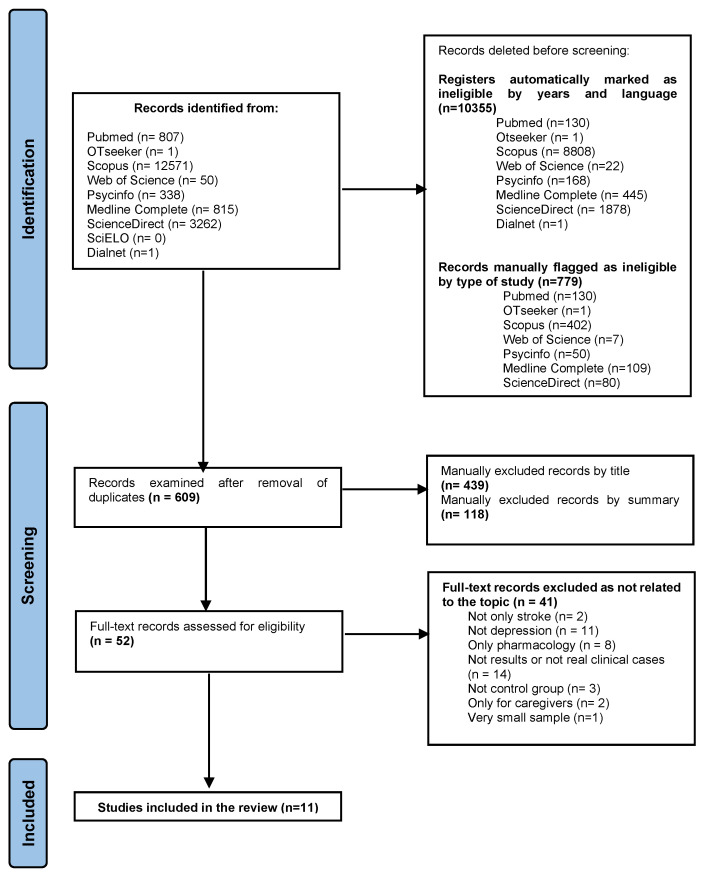
PRISMA Flowchart of search.

To carry out this systematic review, an exhaustive search was performed in the databases: Pubmed, Otseeker, Scopus, Web of Science, Psycinfo, Medline Complete, ScienceDirect, SciELO, and Dialnet to identify controlled clinical trials or randomized clinical trials, published in the last ten years (see Table 2). A figure was developed according to the PRISMA regulation and the trial selection was attached (see Figure 1). The search for all published studies was conducted in December 2021 and updated in May 2022.

The quality assessment of the selected studies was carried out by two independent researchers using PEDro Scale [33].

The full search strategy for all databases was: (‘stroke’ AND ‘depression’ AND ‘treatment outcome’) and the search filters selected were ‘last 10 years’/’2012–2022’, ‘English’, ‘Spanish’. Except for ‘Pubmed’ and ‘OTseeker’ a manual reading was carried out to select those studies that were randomized or controlled clinical trials (Table 2).

### 2.3. Selection Criteria

Only studies that met the following criteria were included in this systematic review: controlled clinical trials or randomized clinical trials, published in the last 10 years, involving women and/or men over 18 years of age who had suffered an ischemic or hemorrhagic stroke and highlighting the change in their psychological state through assessment scales such as the Beck Depression Inventory (BDI), Hamilton Depression Rating Scale (HAM-D) or Hospital Anxiety and Depression Scale (HADS). The languages included were English and Spanish.

Exclusion criteria were: the rest of the types of studies that are not exposed in the inclusion criteria and studies that focused on the psychological state of caregivers and did not focus the research on the psychological state of users were excluded.

## 3. Results

### 3.1. Search Results

In May, the search results were checked after performing the same strategy. Figure 1 was developed according to the PRISMA regulation.

### 3.2. Characteristics of Included Trials

The 11 resulting clinical trials are summarized in Table 3 (see Table 3).

### 3.3. Included Studies

After the search, 779 articles were retrieved and 170 were eliminated as duplicates. Finally, a total of 609 articles were retrieved of which 52 were read in full text. The final sample of the systematic review was composed of 11 clinical trials (see Table 1). The selection of these articles was based on the objectives and the application of the defined inclusion and exclusion criteria. The most relevant information from the articles obtained is summarized in Table 3 (see Table 3).

The identification of included studies was conducted independently in three stages: in the first stage; duplicates were manually removed; in the second stage; the title and abstract were read to select valid studies; and in the third stage; the full text of the resulting studies was assessed.

The following information was extracted independently for all studies, using a template (Table 3): author (main author is written), year of publication, type of study, participants (the total number), the study group (description of the intervention and the sample assigned to each group), characteristics of the intervention (duration of the intervention and the procedure followed by each group), measures (assessment scales used), and outcomes (reflection of the effectiveness of the intervention and possible significant underachievement). At the end of the table, the resulting score after the internal validity analysis (PEDro scale) is presented.

### 3.4. Quality of Trials

To minimize the risk of bias, the PEDro Scale was used to assess the internal validity of the studies in this systematic review. The scoring of the studies is in Table 3. This tool helped to identify randomized clinical trials through 10 items, which assess the specificity of choice criteria, randomization of subjects, blinded allocation, outcome measures, outcome statistics, and variability [45]. It was agreed to score at least five points in the evaluation of the studies to be included in the final review. Table 3 shows how the majority of the studies included have a score above seven, so they have a very good internal validity according to the range of the PEDro scale.

To complete the evidence of this review, the results of randomized clinical trials have to include the Relative Risk (RR). This measure reflects the strength of the association between receiving or not receiving the intervention, indicating if RR > 1 that the intervention is associated with positive recovery outcomes. The measurement of RR is carried out with the following formula [46]:

Table 4 shows the Relative Risk of all included studies (see Table 4):

$$RR=\frac{Intervention\;Group}{Control\;Group}=\frac{I^{IG}}{I^{CG}}=\frac{a/c}{b/c}$$

The sample size of the included studies is a variable that stands out in this systematic review. Excepting the clinical trial by Taravati et al. [36] with *n* = 37, the clinical trial by Olukolade and Osinowo [40] with *n* = 30, and the one by Chun et al. [42] with *n* = 59, the rest presented a sample size equal to or greater than 60 patients.

Regarding the periodicity of the interventions proposed in the included studies, the majority lasted longer than two months. However, four clinical trials had a shorter duration [28,30,31,32].

In terms of assessment instruments, all studies have used multiple standardized tests. For the assessment of depression status, the most commonly used tools have been found to be the Hospital Anxiety and Depression Scale (HADS) [31,32,33,34], the Hamilton Depression Scale (HAM-D) [30,33,35,36], and the Beck Depression Inventory (BDI) [29,30,36]. Other aspects such as occupational performance measured through the assessment instrument known as the Barthel Index (BI) were addressed [31,34,36,37].

Regarding the quality of the article, it was found that they achieved a medium-high score excluding one study. This random clinical trial scored below average because it did not present a method of sample randomization as can be seen in Table 3 [39].

**Figure 2:**
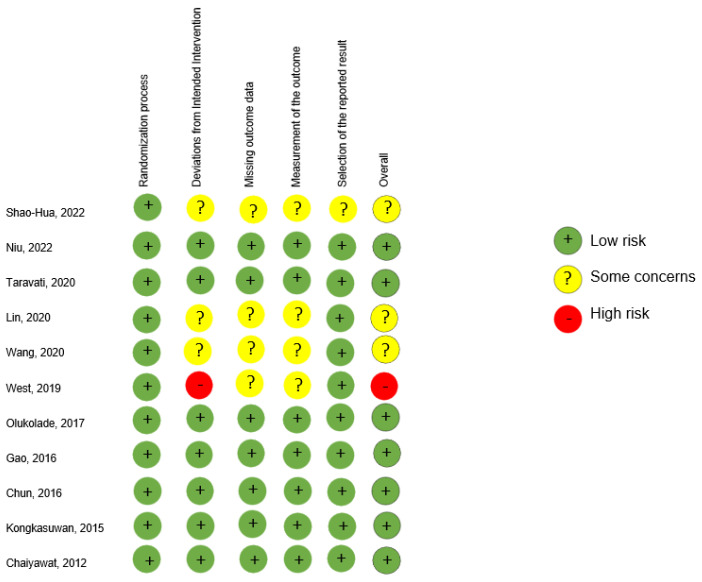
Risk of Bias assessment according to the Cochrane Collaboration’s tool (RoB 2.0) for randomised controlled trials. Higgins et al. (2011) [47].

The Risk of Bias tool by Cochrane Collaboration [47] was used to assess the methodology of scientific evidence of the RCTs included in this systematic review. The risk of bias assessment for each RCT included and by domain is summarized in Figure 2.

The internal validity of the articles corresponded to the scientific evidence shown by the methodology of the included articles. Figure 2 shows how most articles have a low risk of bias. Three studies present a worrying risk of bias because they do not present much information on the domain of the intervention allocation effect and how it affects the outcome measure. Only one study shows a high risk of bias because the assessors were aware of the intervention assigned to the participant and it could affect the context.

## 4. Discussion

The main objective of the present systematic review focused on studying the effect of depression on functional outcomes and long-term recovery after stroke, as shown in the results. Then, the articles were discussed according to how they achieved the objectives of this systematic review.

### 4.1. Impact of Depression on Functionality and Recovery

Olukolade and Osinowo [40], Kongkasuwan et al. [43] and Zhang et al. [34], and Wang et al. [38] highlighted the importance of diagnosing and treating post-stroke depression to improve the rehabilitation process and increasing patients’ quality of life, leading to a greater and more effective recovery.

The daylight intervention in inpatient rehabilitation confirmed an improvement in the psychological state of the person leading to reduced depression and increased well-being at the time of discharge [39].

The results of several interventions (Chun et al. [42], Chaiyawat and Kulkantrakorn [44], and Zhang et al. [34]) showed a reduction in depression and anxiety that resulted in less dysfunctionality and dependency. Furthermore, in the article by Chaiyawat and Kulkantrakorn [44] the high score on the HADS assessment of anxiety and depression during the hospital stay resulted in a lower score on the instrument assessing the performance of activities of daily living (Barthel Index).

Several articles confirmed that depression was strongly associated with dependence and quality of life [28,34,38].

The only clinical trial that conducted a virtual reality intervention combined with physical rehabilitation showed in its results a decrease in the depressive mood of the patients who received it, which increased the functionality and muscle strength of the affected and unaffected physical parts [37].

However, several authors such as Taravati et al. [36], Kongkasuwan et al. [43], West et al. [39], and Niu et al. [35], did not show significant changes in the outcomes of the cognitive aspects of their interventions (as assessed by MoCA) or in the long-term functional prognosis of their interventions. Furthermore, changes in depression were also not confirmed by the single use of antidepressants [41].

Sometimes, over time, symptoms were able to improve on their own by restoring neurological function [35].

### 4.2. Impact of Depression on Women Versus Men

All of the resulting articles included stroke survivors 55 years through 80 years old, of which five were notable for their large female population [29,32,34,35,38]. All were randomized clinical trials except one, which was a quasi-experimental clinical study [44].

Another study highlighted the importance of presenting anxiety and depression after stroke, beyond the physical and cognitive disability [39]. This was presented as a result of the intervention study by Gao et al. [41] where they assessed patients at discharge, at 2–3 months, and at 6–9 months after discharge [41].

Olukolade and Osinowo [40] indicated that it is important to diagnose and treat depression after stroke to benefit patients’ medical condition and quality of life, leading to a reduction in pain and disability and thus greater adherence to treatment.

Mental health may be more vulnerable after a stroke because cognitive impairments (concentration or performance) may interfere with emotional states [36].

### 4.3. The Need for Occupational Therapy Approach in Physical and Mental Rehabilitation

Olukolade and Osinowo highlighted that the type of therapy has a significant influence on post-stroke depression, both at the beginning and at the end of treatment [40].

The discipline of occupational therapy can implement different therapeutic interventions such as the one presented in Kongkasuwan et al. article on creative art therapy [32]. This intervention allowed stroke patients who have suffered a stroke and experienced emotional disturbance with loss of motor skills, to improve their depressive symptoms and quality of life.

Interventions for cognitive training (computer or virtual reality) and learning showed results of improvements in cognitive function, depression, anxiety, sleep quality, and self-care in patients with moderate cognitive states after stroke [29,31,35]. It was even more effective than psychoeducation and the usual care provided [40].

Psychological support and self-awareness therapies insisted on their efficacy in reducing depressive symptoms [30,34,37,38]. Nevertheless, cognitive behavioral therapy was the most effective [41].

The combination of physical rehabilitation, patient education to restore daily living functions, problem-solving skills and counseling made patients satisfied and it reduced the feeling of lack of support that they experienced after hospital discharge [44].

In all the trials reviewed, no significant changes were found with the single use of pharmacological therapies. In contrast, the results of a citalopram intervention highlighted the adverse effects of long-term medication [30,36].

## 5. Limitations

There are heterogeneous studies in the methodology (number of patients, type of therapy, and duration). The percentage of the sample whose results were striking did not correspond to the original sample size.

The occupational therapy approach has not been studied in depth due to the fact that this profession has difficulties to been recognized in the hospital environment [38,42,45,48].

## 6. Conclusions

Based on all the points discussed in this systematic review, it can be concluded that post-stroke rehabilitation should include the prevention of depression to allow greater recovery of functionality and to improve quality of life.

According to the second objective, it is shown that post-stroke depressive disorder may differ by gender. Depressive symptoms are common after stroke and the female gender is associated with them, resulting in a higher level of disability and poorer quality of life.

Therefore, it is necessary to attend to the individuality of the person and to offer treatment according to the different aspects surrounding them (gender, age, background, and family support...). All of this could speed up functional recovery and improve the patient’s and their family’s quality of life.

Finally, it is important to explore more studies that research the effects of occupational therapy practice on physical and mental recovery in post-stroke patients with depression. There are very little scientific data to support this hypothesis, but the results of the various studies are consistent in showing improvements in all areas that an occupational therapist’s knowledge and practice can deal with.

## Figures and Tables

**Table 1:** Search strategy.

Database	Search Strategy	Results
Pubmed	((“stroke”) AND (“depression”)) AND (“treatment outcome”)	807
Otseeker	[Any Field] like ‘“stroke”‘ AND [Any Field] like ’”depression”‘ AND [Any Field] like ’”treatment outcome”‘	1
Scopus	“stroke” AND ”depression” AND ”treatment outcome”	12,571
Web of Science	((ALL = (“stroke”)) AND ALL = (“depression”)) AND ALL = (“treatment outcome”)	50
Psycinfo	Stroke AND ”depression” AND ”treatment outcome”	338
Medline Complete	“stroke” AND ”depression” AND ”treatment outcome”	815
Science Direct	Stroke AND ”depression” AND ”treatment outcome”	3262
SciELO	“stroke” and “depression” and ”treatment outcome”	0
Dialnet	Stroke AND ”depression” AND ”treatment outcome”	1
		17,845

**Table 2:** Final strategy of searching in Databases with filters.

Database	Search Filters	Final Strategy	Results
Pubmed	Randomized Controlled Trial, in the last 10 years, English, Spanish	(“stroke” [All Fields] AND “depression” [All Fields] AND “treatment outcome” [All Fields]))	130
OTseeker	without filters	[AnyField] like ’”stroke”‘ AND [Any Field] like ’”depression”‘ AND [Any Field] like ’”treatment outcome”‘	1
Scopus	Limit-to2012– 2022, English, Spanish	“stroke” AND “depression” AND “treatment outcome”	402
Web of Science	Publication Years: 2012– 2022, English	((ALL = (“stroke”)) AND ALL = (“depression”)) AND ALL = (“treatment outcome))	7
Psycinfo	2012–2022, English	“stroke” AND ”depression” AND ”treatment outcome”	50
Medline Complete	Limit to 2012-2022 English	“stroke” AND “depression” AND “treatment outcome”	109
Science Direct	2012–2022	stroke AND ”depression” AND ”treatment outcome”	80
Dialnet	without filters	stroke AND ”depression” AND “treatment outcome”	0
		TOTAL	779

**Table 3:** Summary of the results.

Author/Year	Study Type	Population	Study Group	Intervention Characteristics	Measures	Results	PEDro Scale
Shao-hua, Zhang et al. (2022) [34]	Multicentre Random iZed Controlled Trial	*n* = 660	Patients with cognitive disorders after stroke divided into IDSA group (interactive dynamic scalp acupuncture; *n* = 218), SSCT group (simple combined therapy; *n* = 222) and TSA group (traditional scalp acupuncture; *n* = 220).	2 months (all received conventional medication and physical rehabilitation. The IDSA group received extra computer-based cognitive training).	MoCA, MMSE, HAMD, HAMA, PSQI, MBI	For the SCT and TSA groups, MMSE and MoCA scores were higher. For the IDSA group, HAMA, PSQI and HAMD scores were lower from the second month onward. This group improved in dysfunctionality and dependence (*p* < 0.01)	8/10
Niu, Yunlian et al. (2022) [35]	Randomized Controlled Trial	*n* = 104	Stroke patients receiving G-ACT psychological therapy *n* = 52) versus the group receiving usual care only *n* = 52).	Evaluation of the intervention at 2 weeks, 1 month, and 3 months (5 sessions of 45–50 min of therapy with a specialist during hospitalization).	HAMD, NIHSS, BI	Depressive symptoms decreased in the intervention group, as did the IB score (*p* = 0.018). However, at 3 months the IB measures were similar between groups, so the intervention did not improve the prognosis for functionality (*p* = 0.191)	7/10
Taravati, Sahel et al. (2021) [36]	Blinded Randomized Controlled Study	*n* = 37	Patients with hemiplegia due to stroke divided into a study group with conventional rehabilitation and a robotic-based rehabilitation system (*n* = 17) and a control group with only conventional rehabilitation (*n* = 20).	4 weeks (5 days a week for each type of rehabilitation).	Fulg-Meyer, MoCA, CES- Depression questionnaire	The decrease in depression and the increase in hand strength and function of the patients in the study group are noteworthy (*p* < 0.05) Both groups improved the respective MoCA and Fulg- Meyer	9/10
Lin, Ruei-Ching et al. (2020) [37]	Randomized Controlled Trial	*n* = 143	Hospitalized stroke patients ( *n*= 38) received 5 extra days of virtual reality training.	21 days (5 sesh/week of early rehabilitation; 2 times/day)	MRCScale, HADS, PASS, BI	The experimental group increased muscle strength along with anxiety (*p* = 0.003) and depression (*p* = 0.001).	9/10
Wang, Xiaoyu et al. (2020) [38]	Randomized Controlled Trial	*n*= 134	Patients who had suffered an intracranial hemorrhage received mindfulness-based cognitive therapy (*n* = 67) versus the control group (*n* = 67).	8 weeks (2-h group sessions based on adaptive thoughts, feelings, and experiences).	CES-Dq, MAAS, FACT-Br, NIHSS	In the intervention group, significant changes were found in depression (*p* > 0.01), mindfulness (*p* < 0.01), social and emotional well-being (*p* < 0.05), and quality of life (*p* < 0.05).	8/10
West, Anders et al. (2019) [39]	Quasi-Randomized Controlled Trial	*n* = 71	Stroke patients in a rehabilitation unit equipped with daylight (*n* = 39) versus stroke patients in a rehabilitation unit with standard indoor lighting (*n* = 32).	1 year (simulation of the rhythm of natural light 24 h, being dim until 07:00, maximum until 15:00, and dimmed until 22:00 when it was switched off).	HAMD, HADS, WHO-5, MoCA	The group exposed to natural light had a significant decrease in post-stroke depression and an increase in well-being at discharge (*p* = 0.046). Post-stroke anxiety was also reduced (*p* = 0.045). There were no significant differences in cognition.	5/10
Olukolade, Olugbemi. Osinowo, Helen O. (2017) [40]	Randomized Controlled Trial	*n* = 30	Patients with first stroke divided into groups of *n* = 10 according to the different interventions: group A (cognitive rehabilitation therapy); group B (psychoeducation); group C (usual care).	3.5 months (group A: 9 sessions; group B: 9 sessions; group C: waiting list for care).	BDI, LESS	Cognitive rehabilitation therapy has a greater effect on post-stroke depression (*p* < 0.001) than psychoeducation and usual care. It is suggested that the type of intervention is influential in detecting post-stroke depression both at baseline and after treatment.	9/10
Gao, Jie et al. (2016) [41]	Blinded Randomized Controlled Trial	*n* = 274	A: both placebo medication and psychological therapy (*n* = 91); B: citalopram and psychological therapy with a non-specialist physician (*n* = 91); C: placebo medication and psychological therapy with a specialist (*n* = 92).	3 months (A: discussions twice a week; B: discussions twice a week; C: 2 h of session per week over the 3 months). Sessions of 1 h of Bobath Therapy were held 4/week.	HAMD, MES, BI, BDI, MS, FIM,	Group B improved in depression and melancholy (*p* < 0.05). In fact, this same group had greater adverse effects due to the drug received.	8/10
Chun, Min-Ho et al. (2016) [42]	Randomized Controlled Trial	*n* = 59	Patients with chronic stroke were included in forest group (*n* = 30) versus urban group (*n* = 29).	4 days and 3 nights (the forest group worked on meditation in the forest while the urban group stayed in a hotel).	BDI, HAMD, STAI	The forest group showed a decrease in BDI, HAMD, and SATI scores (*p* < 0.05). See the need to manage psychological conditions (anxiety and depression) in this population.	9/10
Kongkasuwan, Ratcharinetal (2015) [43]	Randomized Controlled Trial	*n* = 118	Stroke patients (older than 50 years old) received only conventional physical therapy (*n* = 59) versus patients who received conventional physical therapy and creative art therapy (*n* = 59).	4 weeks (20 sessions of conventional physical therapy and 8 sessions of creative art therapy of 1.5–2 h in groups of 5–10 patients).	AMT, MBI, HADS, PTQoLq	The creative art therapy group improved functionality, depression (*p* < 0.001), and quality of life (*p* < 0.001) after the intervention. There were no significant differences in anxiety and mental state (*p* = 0.123).	9/10
Chaiyawat, Pakaratee. Kulkantrakorn, Kongkiat. (2012) [44]	Randomized Controlled Trial	*n* = 60	Stroke patients received home exercise program (*n* = 30) versus the control group who only received home rehabilitation instructions (*n* = 30).	6 months (intervention group receives physiotherapy, education, counseling versus problem-solving).	BI, HADS, TMSE	Barthel (*p* < 0.03) and HADS (*p* < 0.01) scores improved after 2 years. Depression was associated with dependency and quality of life. The higher HADS score of the control group showed a lower BI score.	9/10

Legend (measures): MoCA = Montreal Cognitive Assesstment; MMSE = Mini-Mental State Examination; HAMD = Hamilton Depression Rating Scale; HAMA = Hamilton Anxiety Rating Scale; PSQI = Pittsburgh Sleep Quality Index; MBI = Maslach Burnout Inventory; NIHSS = National Institutes of Health Stroke Scale; BI = Barthel Index; Ful-Meyer = Fulg-Meyer; CES-Dq = CES-D Depression Scale; MRCScale = Medical Research Council Manual Muscle Testing Scale; HADS = Hospital Anxiety and Depression Scale; PASS = Postural Assessment Scale for Stroke; MAAS = Mindful Attention Awareness Scale; FACT-Br = Quality of life measurement; WHO-5 = Who-Five well-being Index; BDI = Beck Depression Inventory; LESS = Life Event Stress Scale; MES = Melancholia Scale; MS = Mental Scale; FIM = Functional Independence Measure Scale; STAI = Spielberger State-Train Anxiety Inventory (STAI); AMT = Abbreviated Mental Test (AMT); PTQoLq = Pictorial Thai Quality of Life questionnaire; TMSE = ThaiMini-Mental State Examination.

**Table 4:** Relative Risk.

	Nº Subjects			Measures		
Source, Year	Intervention Group (a)	Control Group (b)	TOTAL (c)	I^IG(a)^	I^CG(b)^	RR
Shao-hua, Zhang et al. (2022)	218 + 222 *	220	660	0.33	0.33	1
Niu, Yunlian et al. (2022	52	52	104	0.5	0.5	1
Taravati, Sahel et al. (2021)	17	20	37	0.46	0.54	0.85
Lin, Ruei-Ching et al. (2020)	38	107	143	0.26	0.75	0.35
Wang, Xiaoyu et al. (2020)	67	67	134	0.64	0.64	1
West, Anders et al. (2019)	39	32	71	0.55	0.63	0.87
Olukolade, Olugbemi. Osinowo, Helen O. (2017)	10 + 10 *	10	30	0.33	0.33	1
Gao, Jie et al. (2016)	91 + 92 *	91	274	0.33	0.33	1
Chun, Min-Ho et al. (2016)	30	20	59	0.51	0.34	1.5
Kongkasuwan, Ratcharin et al. (2015)	59	59	118	0.5	0.5	1
Chaiyawat, Pakaratee. Kulkantrakorn, Kongkiat. (2012)	30	30	60	0.5	0.5	1

* There are two similar groups of intervention versus one control group.
